# Supplementary material for: Enhanced Third‐Harmonic Generation in a Bound State in the Continuum Assisted Multiband All‐Dielectric Metasurface
Source: Nanophotonics. 2026 Nov 10;15(21):e70346. doi: 10.1002/nap2.70346 (PMC13752562; doi:10.1002/nap2.70346)
Supplement: Supplementary file 1 [file NAP2-15-e70346-s001.pdf]

Supplementary Material: Enhanced Third-Harmonic Generation in a Bound State in the Continuum Assisted Multiband All-Dielectric Metasurface

Partha Mondal[1], Hadeel Alamoudi[2], Rodrigo P. Sanchez[2], Redha H. Al Ibrahim[1], Boon S. Ooi[1], Iman Roqan[2], and Hakan Bagci[1]

[1]Electrical and Computer Engineering (ECE) Program
Computer, Electrical and Mathematical Science and Engineering (CEMSE) Division
King Abdullah University of Science and Technology (KAUST)
Thuwal, 23955-6900, Saudi Arabia

[2]Material Science and Engineering (MSE) Program
Physical Science and Engineering (PSE) Division
King Abdullah University of Science and Technology (KAUST)
Thuwal, 23955-6900, Saudi Arabia

*Corresponding author: Partha Mondal (partha.mondal@kaust.edu.sa)

1 Multipolar decomposition

The scattering response of the metasurface is analyzed using a Cartesian multipole decomposition, in which the total scattering cross section is written as the sum of the contributions from the electric dipole (ED), toroidal dipole (TD), magnetic dipole (MD), electric quadrupole (EQ), and magnetic quadrupole (MQ) moments [1–3],

$$\begin{aligned} C_{\mathrm{sca}} &= C_{\mathrm{sca}}^{\mathrm{ED}} + C_{\mathrm{sca}}^{\mathrm{TD}} + C_{\mathrm{sca}}^{\mathrm{MD}} + C_{\mathrm{sca}}^{\mathrm{EQ}} + C_{\mathrm{sca}}^{\mathrm{MQ}} + \cdots \\ &= \frac{k^4}{6\pi\varepsilon_0^2|\mathbf{E}_{\mathrm{inc}}|^2}\left[\sum_{\alpha}\left(|p_\alpha + ik\,T_\alpha|^2 + \frac{|m_\alpha|^2}{c}\right) + \frac{1}{120}\sum_{\alpha\beta}\left(|kQ^e_{\alpha\beta}|^2 + \left|\frac{kQ^m_{\alpha\beta}}{c}\right|^2\right) + \cdots\right], \end{aligned}$$

where p_α and T_α are the electric and toroidal dipole moments, m_α is the magnetic dipole moment, and $Q^e_{\alpha\beta}$ and $Q^m_{\alpha\beta}$ are the electric and magnetic quadrupole moments, respectively. Here, $\alpha, \beta \in \{x, y, z\}$ are Cartesian indices, $\mathbf{J}$ is the induced current density with component J_α, $\mathbf{r}$ is the position vector with component r_α and magnitude r, ω is the angular frequency, $\delta_{\alpha\beta}$ is the Kronecker delta, $|\mathbf{E}_{\mathrm{inc}}|$ is the amplitude of the incident plane-wave field, k is the wavenumber, c is the speed of light, and ε_0 is the vacuum permittivity. The corresponding multipole moments are given by [1]

$$p_\alpha \approx -\frac{1}{i\omega}\int J_\alpha\,\mathrm{d}^3\mathbf{r},$$

$$T_\alpha \approx \frac{1}{10c}\int\left\{(\mathbf{r}\cdot\mathbf{J})r_\alpha - 2r^2 J_\alpha\right\}\mathrm{d}^3\mathbf{r},$$

$$m_\alpha \approx \frac{1}{2}\int(\mathbf{r}\times\mathbf{J})_\alpha\,\mathrm{d}^3\mathbf{r},$$

$$\begin{aligned} Q^e_{\alpha\beta} \approx -\frac{1}{i\omega}\Bigg[&\int\left\{3(r_\beta J_\alpha + r_\alpha J_\beta) - 2(\mathbf{r}\cdot\mathbf{J})\delta_{\alpha\beta}\right\}\mathrm{d}^3\mathbf{r} \\ &+ \frac{k^2}{4}\int\left\{4r_\alpha r_\beta(\mathbf{r}\cdot\mathbf{J}) - 5r^2(r_\alpha J_\beta + r_\beta J_\alpha) + 2r^2(\mathbf{r}\cdot\mathbf{J})\delta_{\alpha\beta}\right\}\mathrm{d}^3\mathbf{r}\Bigg], \end{aligned}$$

$$Q^m_{\alpha\beta} \approx \int \Big\{ r_\alpha (\mathbf{r} \times \mathbf{J})_\beta + r_\beta (\mathbf{r} \times \mathbf{J})_\alpha \Big\} \, \mathrm{d}^3\mathbf{r}.$$

Here, p_α denotes the leading electric dipole term, and the toroidal dipole moment T_α, retained beyond the long-wavelength approximation, is treated as a separate scattering channel; the two contribute jointly through the combination $p_\alpha + ikT_\alpha$ in the cross section.

2 Fano fitting: Simulated transmission spectra

The simulated transmission spectra at each resonance are fitted with the Fano line-shape model to extract the corresponding Q factors. Figure S1 shows the fitted spectra and provides the extracted Q factors for the eight resonances M_1–M_8 at $\delta = 0.14$

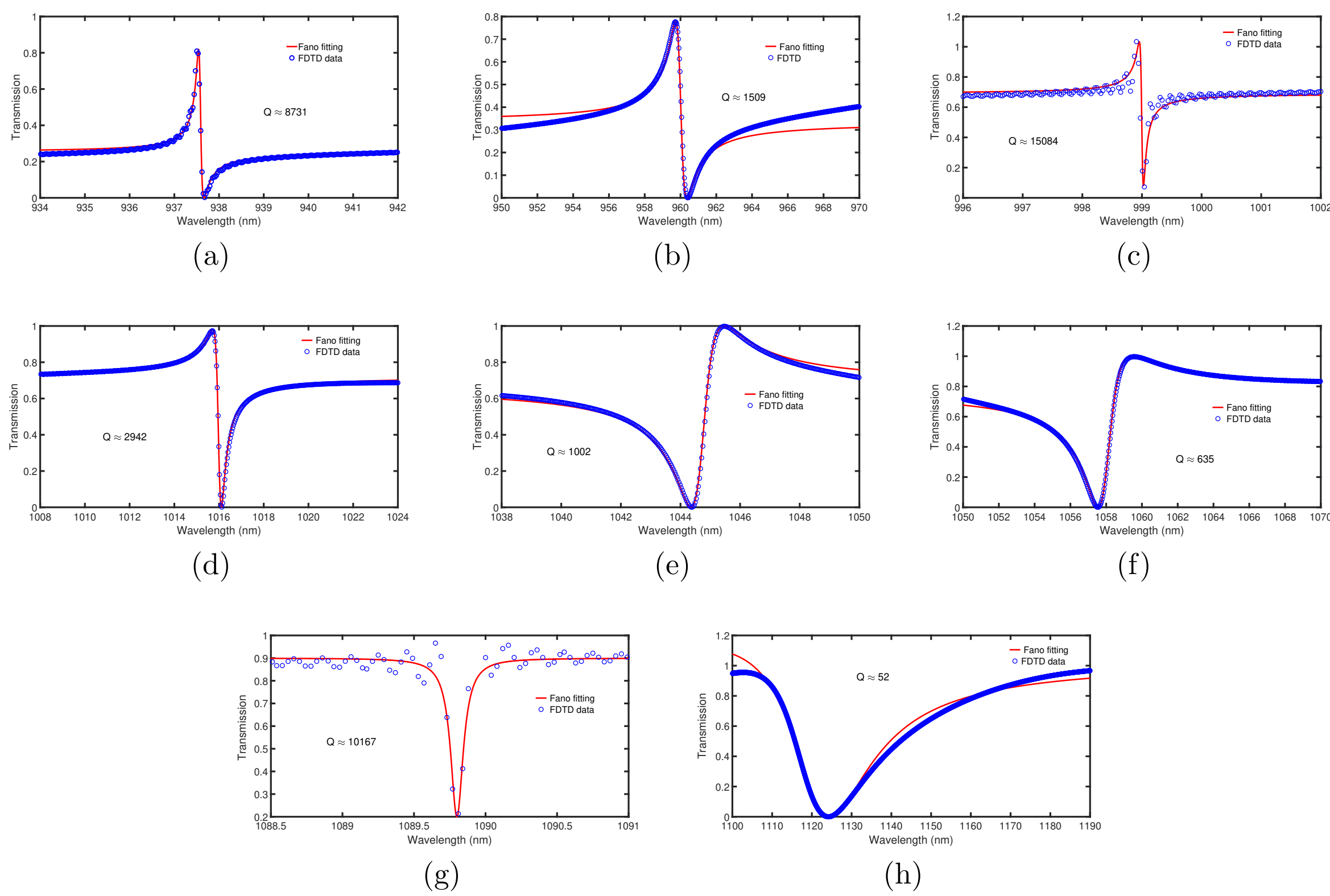

Figure S1: Fano fitting of the simulated transmission spectra at $\delta = 0.14$ for the resonances (a) M_1 ($Q = 8\,731$), (b) M_2 ($Q = 1\,509$), (c) M_3 ($Q = 15\,084$), (d) M_4 ($Q = 2\,942$), (e) M_5 ($Q = 1\,002$), (f) M_6 ($Q = 635$), (g) M_7 ($Q = 10\,167$), and (h) M_8 ($Q = 52$).

3 Field distribution

The electric and magnetic field distributions at each resonance are examined to identify the modes underlying the observed resonances. Figure S2 shows the simulated electric field distributions in the xz-plane, and Figure S3 shows the magnetic field distributions in the xy-plane, all at $\delta = 0.14$ for the eight resonances M_1–M_8.

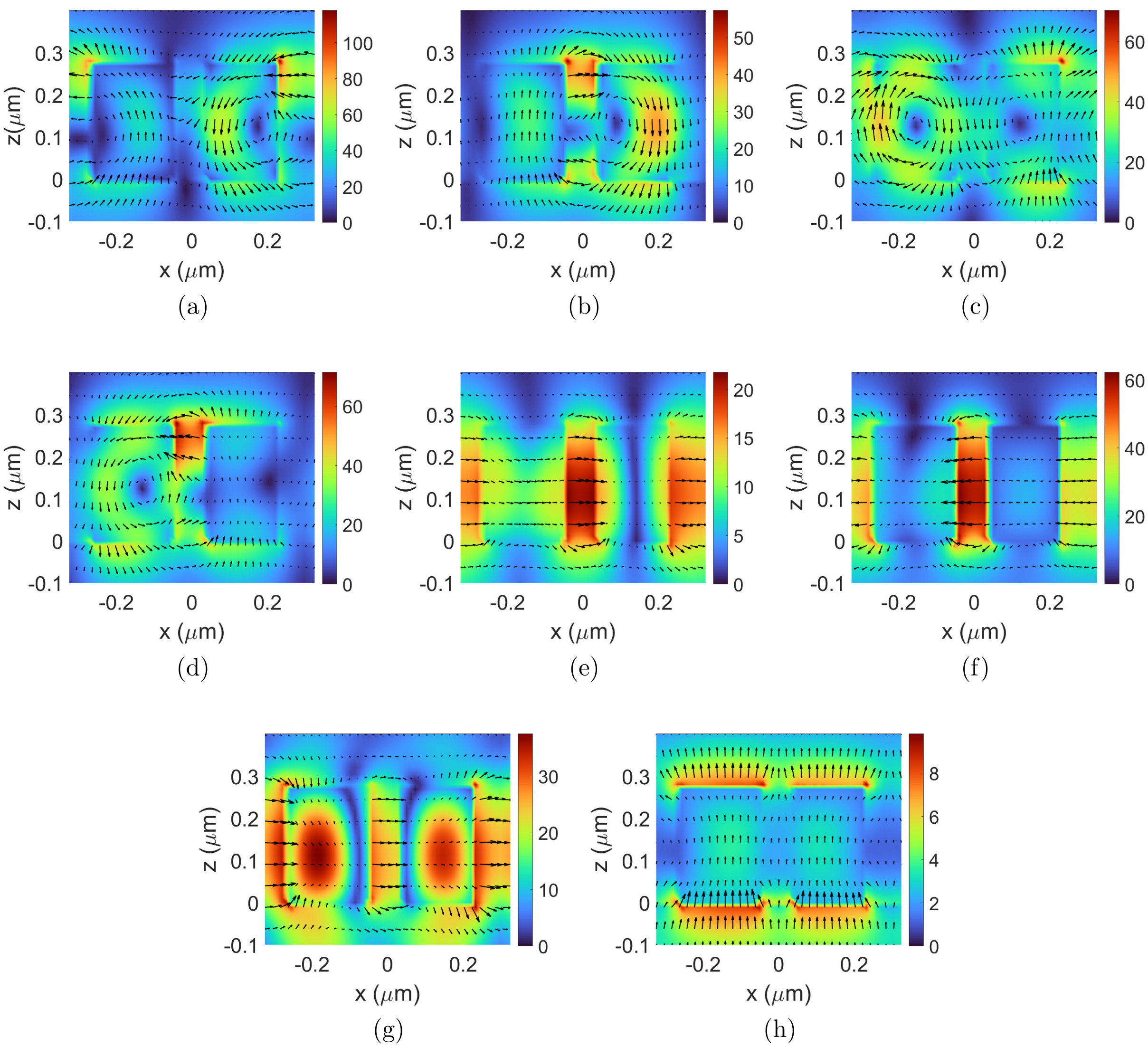

Figure S2: Simulated electric field distributions in the xz-plane at $\delta = 0.14$ for the resonances (a) M_1, (b) M_2, (c) M_3, (d) M_4, (e) M_5, (f) M_6, (g) M_7, and (h) M_8.

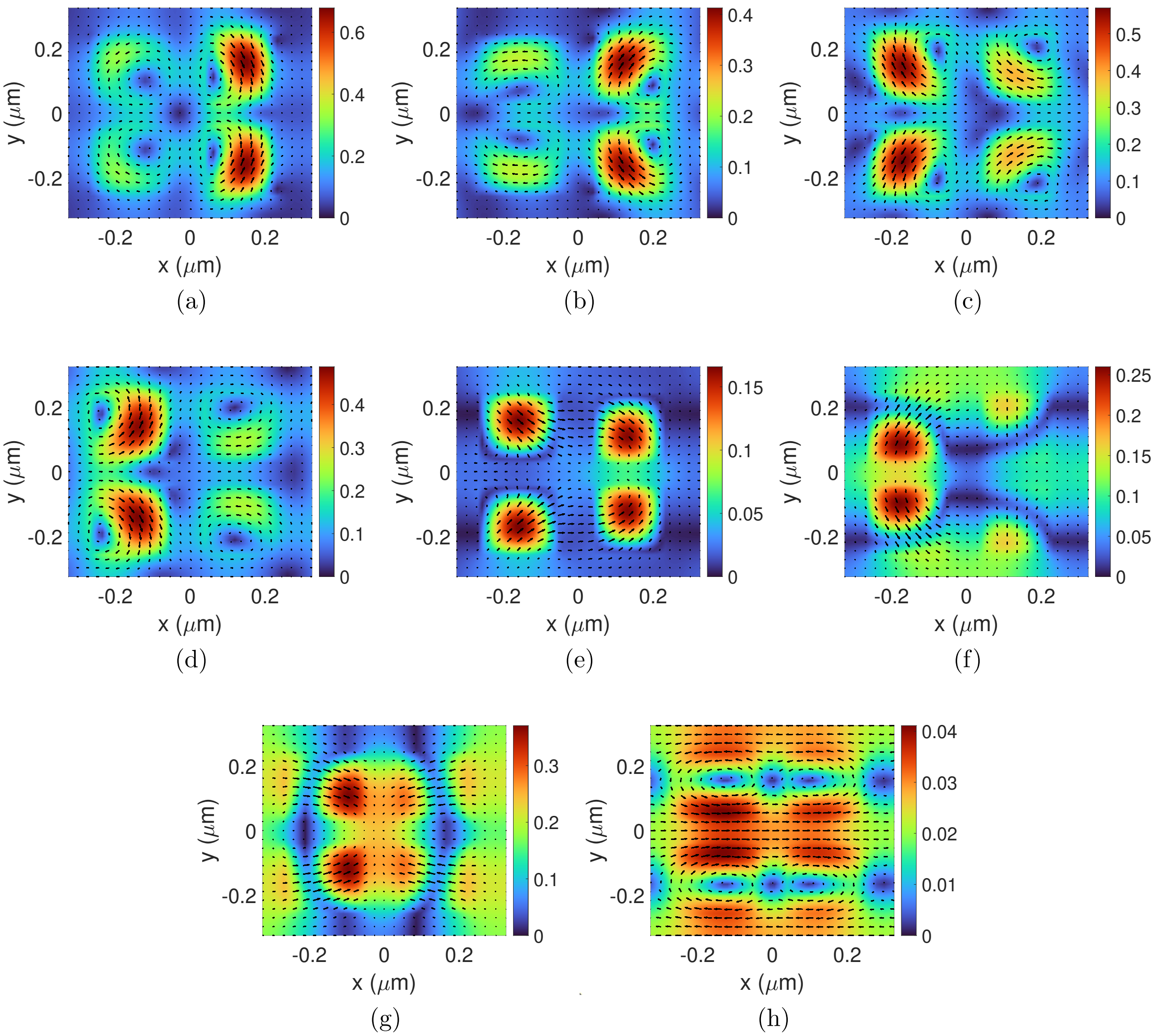

Figure S3: Simulated magnetic field distributions in the xy-plane at $\delta = 0.14$ for the resonances (a) M_1, (b) M_2, (c) M_3, (d) M_4, (e) M_5, (f) M_6, (g) M_7, and (h) M_8.

4 TH conversion efficiency

Nonlinear simulations are performed to evaluate the TH response at each resonance. Figure S4 shows the simulated conversion efficiencies of the TH spectra for the eight resonances M_1–M_8 at $\delta = 0.14$.

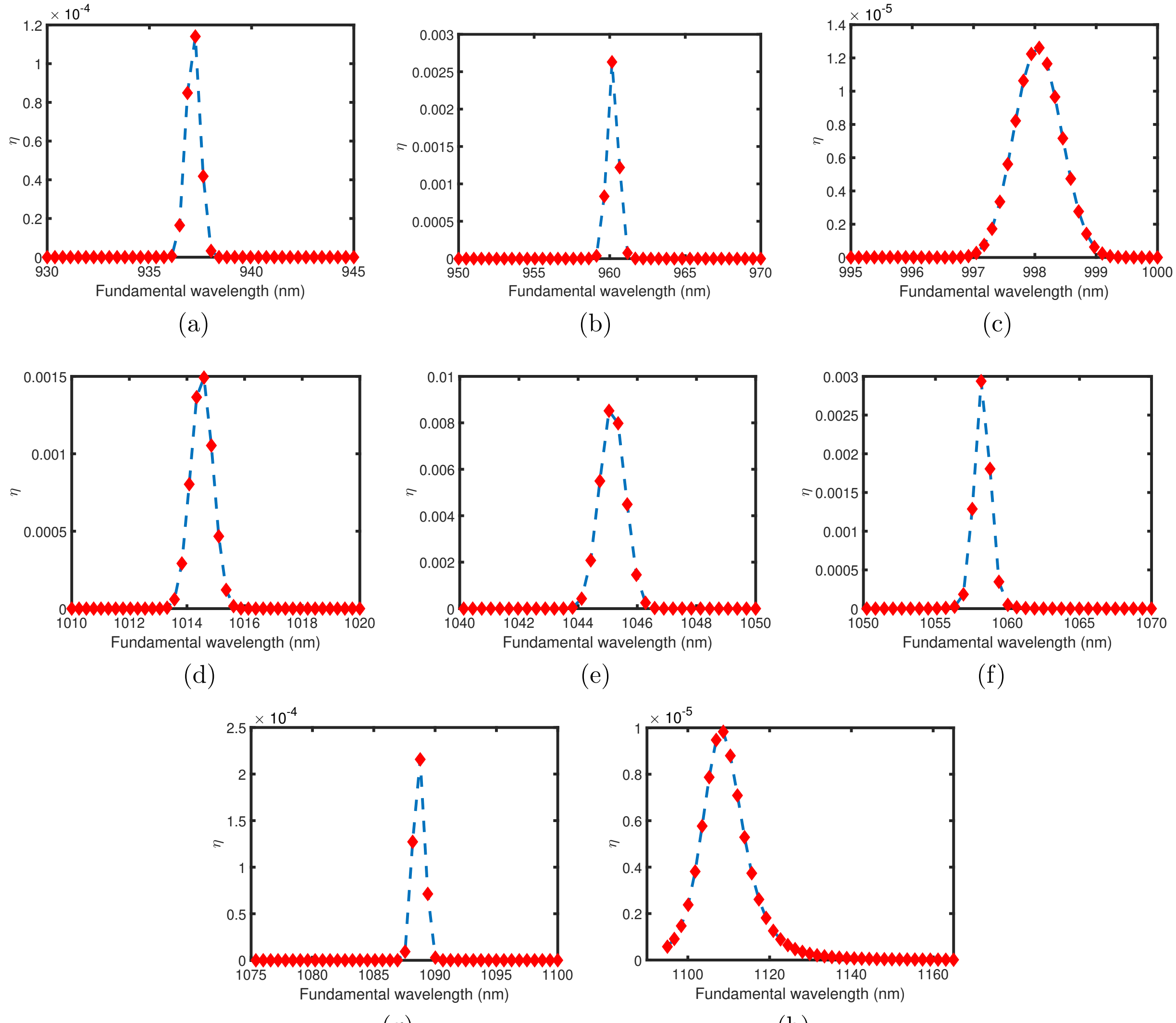

Figure S4: Simulated conversion efficiency as a function of pump wavelength across the multiband resonances, computed at $\delta = 0.14$, peak pump intensity $1.6\,\mathrm{GW/cm^2}$, and effective pump-beam area $314\,\mu\mathrm{m}^2$. The plotted conversion efficiencies correspond to excitation at the resonances (a) M_1, (b) M_2, (c) M_3, (d) M_4, (e) M_5, (f) M_6, (g) M_7, and (h) M_8.

5 Nonlinear fitting

The third-order nature of TH generation is confirmed by the power dependence of the simulated TH power. Figure S5 shows a log–log plot of the simulated TH power versus average input pump power for the M_8 resonance.

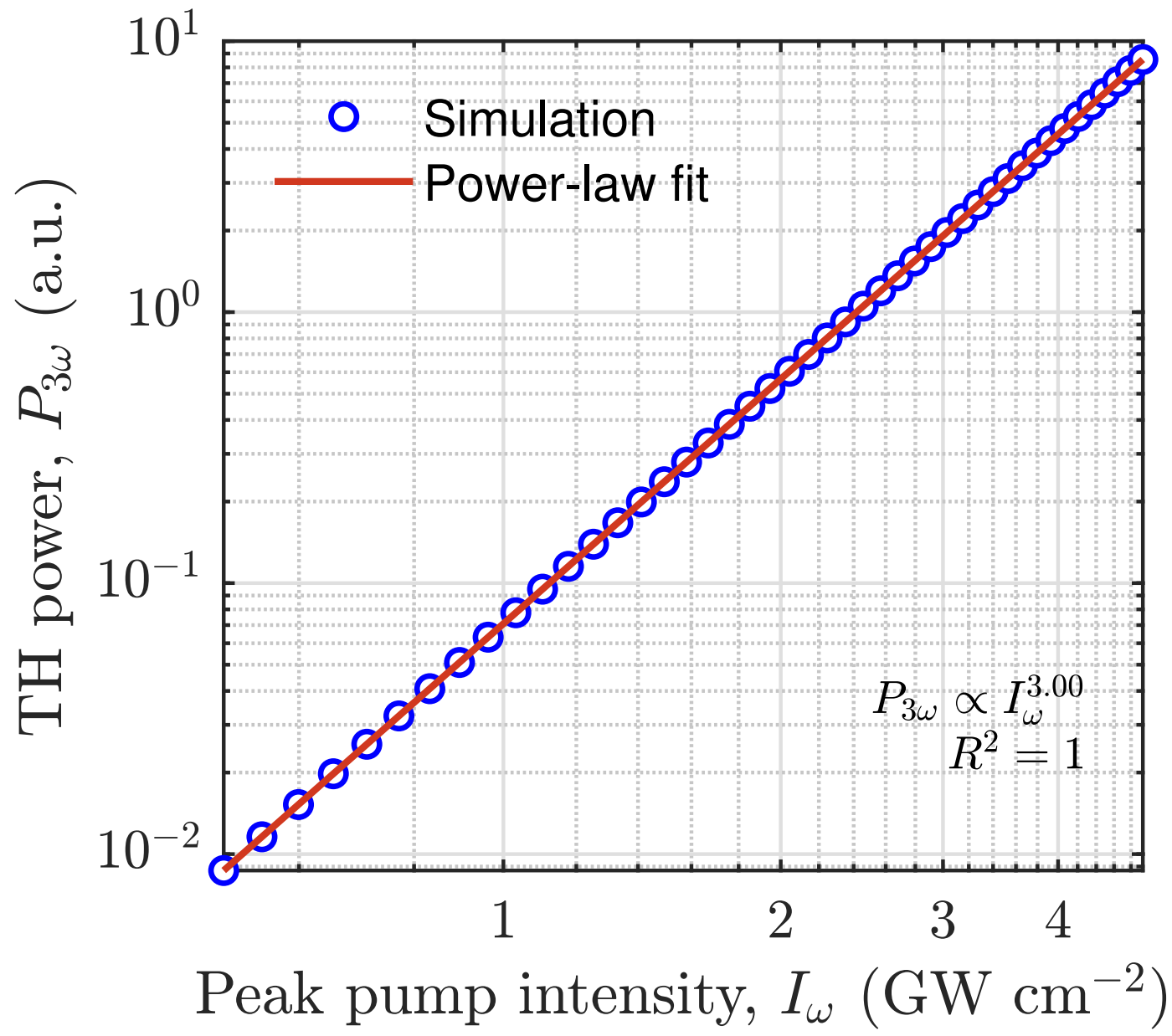

Figure S5: Simulated TH power $P_{3\omega}$ as a function of the peak pump intensity I_ω at the M_8 resonance, plotted on a log–log scale. The solid line represents the power-law fit $P_{3\omega} = a\,(I_\omega)^b$, yielding $a = 0.07082$, $b = 3.00$, and $R^2 = 1$. The cubic dependence confirms the third-order nonlinear origin of the generated TH signal.

6 Device fabrication

The metasurface is fabricated using standard thin-film deposition and electron-beam lithography (EBL). Figure S6 shows the main fabrication steps.

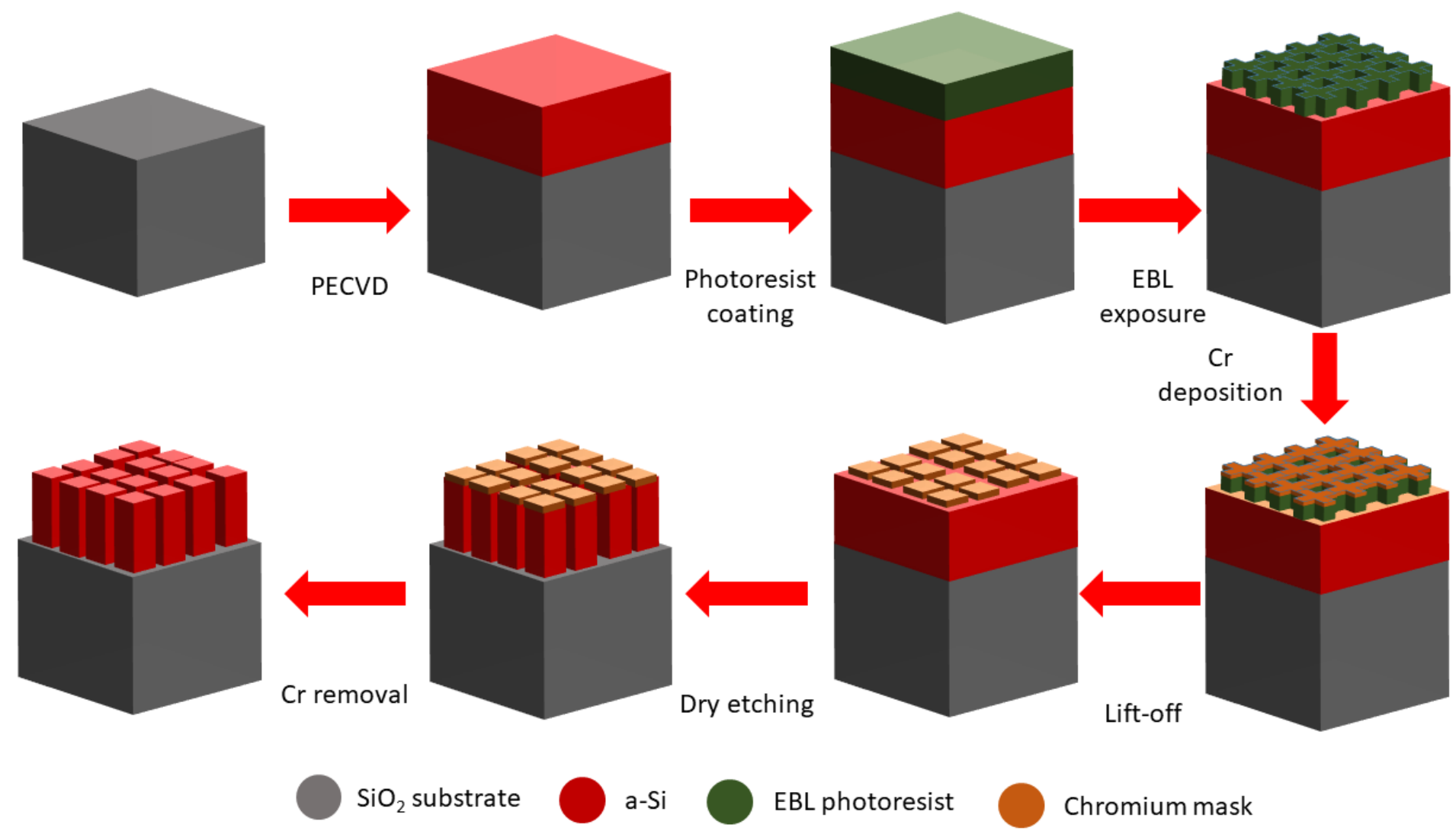

Figure S6: Fabrication steps of the all-dielectric metasurface. An amorphous silicon (a-Si) layer is deposited onto a glass substrate using plasma-enhanced chemical vapor deposition (PECVD). The designed metasurface patterns are then defined in a thin layer of positive photoresist (AR-P 6200) by EBL. A 20 nm-thick chromium (Cr) film is deposited by electron-beam evaporation to serve as a hard etching mask, and lift-off leaves the mask defining the pattern. Finally, reactive ion etching (RIE) transfers the pattern into the a-Si layer, and the chromium mask is subsequently removed.

7 Fano fitting: Measured transmission spectra

The measured transmission spectra at each resonance are fitted with the Fano line-shape model to extract the corresponding Q factors. Figure S7 shows the fitted spectra and provides the extracted Q factors for the eight resonances $M_{1\mathrm{x}}$–$M_{8\mathrm{x}}$ at $\delta = 0.14$.

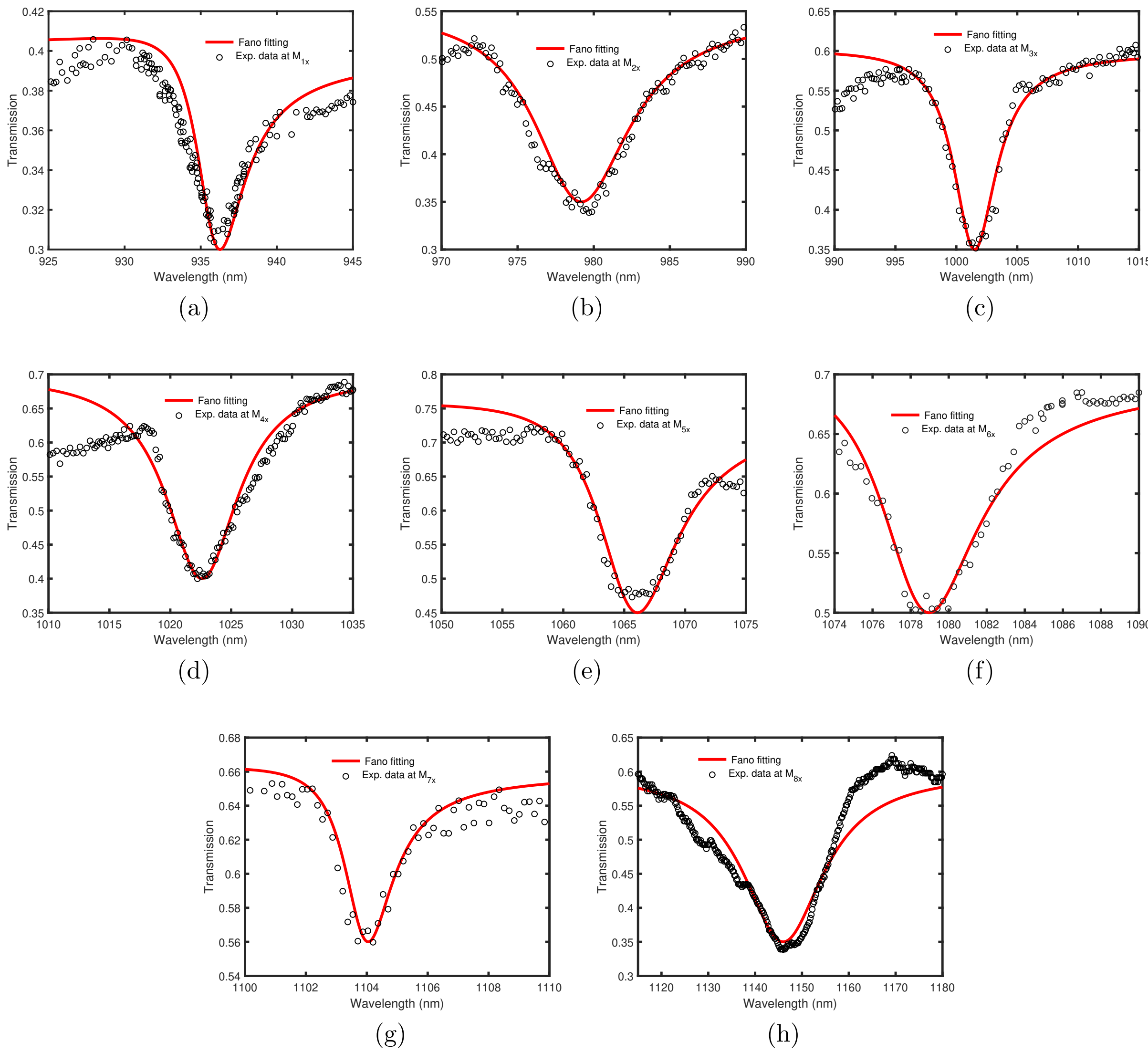

Figure S7: Fano fitting of the measured transmission spectra at $\delta = 0.14$ for the resonances (a) M_{1x} ($Q = 271$), (b) M_{2x} ($Q = 148$), (c) M_{3x} ($Q = 260$), (d) M_{4x} ($Q = 161$), (e) M_{5x} ($Q = 156$), (f) M_{6x} ($Q = 195$), (g) M_{7x} ($Q = 550$), and (h) M_{8x} ($Q = 51$).

References

[1] Rasoul Alaee, Carsten Rockstuhl, and Ivan Fernandez-Corbaton. An electromagnetic multipole expansion beyond the long-wavelength approximation. *Optics Communications*, 407:17–21, 2018.

[2] Kseniia V Baryshnikova, Daria A Smirnova, Boris S Luk'yanchuk, and Yuri S Kivshar. Optical anapoles: concepts and applications. *Advanced Optical Materials*, 7(14):1801350, 2019.

[3] Tatsuki Hinamoto and Minoru Fujii. Menp: an open-source matlab implementation of multipole expansion for nanophotonics. *Osa Continuum*, 4(5):1640–1648, 2021.
